# Stenting with dual-layer CGuard stent in acute sub-occlusive carotid artery stenosis and in tandem occlusions: a monocentric study

**DOI:** 10.1007/s00234-024-03397-w

**Published:** 2024-06-07

**Authors:** Mousa Zidan, Christian Gronemann, Nils Christian Lehnen, Felix Bode, Johannes Weller, Gabor Petzold, Alexander Radbruch, Daniel Paech, Franziska Dorn

**Affiliations:** 1 Department of Neuroradiology, Bonn University Hospital, Venusberg-Campus1, Gebäude 81, 53127 Bonn, Germany; 2 Department of Neurology, Bonn University Hospital, Bonn, Germany; 3 Department of Radiology, German Cancer Research Centre, Heidelberg, Germany; 4 Department of Neuroradiology, LMU-Klinikum der Universität München Medizinische Klinik und Poliklinik IV, Munich, Bayern, Germany

**Keywords:** Carotid artery stenting, Tandem occlusion, Dual-layer stent, Thrombectomy, In-stent occlusion

## Abstract

**Purpose:**

Double-layer design carotid stents have been cast in a negative light since several investigations reported high rates of in-stent occlusions, at least in the acute setting of tandem occlusions. CGuard is a new generation double-layered stent that was designed to prevent periinterventional embolic events. The aim of this study was to analyze the safety and efficacy of the CGuard in emergent CAS and for the acute treatment of tandem occlusions in comparison with the single-layer Carotid Wallstent (CWS) system.

**Methods:**

All patients who underwent CAS with CGuard or CWS after intracranial mechanical thrombectomy (MT) between 11/2018 and 12/2022 were identified from our local thrombectomy registry. Clinical, interventional and neuroimaging data were analyzed. Patency of the stent was assessed within 72 h. Intracranial hemorrhage and modified Rankin score (mRS) at discharge were the main endpoints.

**Results:**

In total, 86 stent procedures in 86 patients were included (CWS: 44, CGuard: 42). CGuard had a lower, but not statistically significant rate (*p* = 0.431) of in-stent occlusions (*n* = 2, 4.8%) when compared to the CWS (*n* = 4, 9.1%). Significant in-stent stenosis was found in one case in each group. There was no statistically significant difference in functional outcome at discharge between the two groups with a median mRS for CGuard of 2 (IQR:1–5) vs. CWS 3 (IQR:2–4).

**Conclusion:**

In our series, the rate of in-stent occlusions after emergent CAS was lower with the dual-layer CGuard when compared to the monolayer CWS. Further data are needed to evaluate the potential benefit of the design in more detail.

## Introduction

Mechanical thrombectomy (MT) has established itself as the best possible treatment in the setting of an acute ischemic stroke (AIS) [1]. Up to 15% of all patients with a large vessel occlusion (LVO) in the anterior circulation also have an extracranial occlusion or high-grade stenosis [2, 3]. Tandem occlusions (TO) are associated with an increased risk for intracranial hemorrhage (ICH) and have in general an unfavorable prognosis [4]. In a setting of TO, intracranial treatment is frequently performed first to hasten early brain reperfusion and then acute carotid artery stenting (aCAS) is carried out [5, 6]. In instances of acutely symptomatic thrombogenic sub-occlusive lesions affecting the extracranial internal carotid artery ICA, stenting is frequently employed to address the specific lesion and prevent the recurrence of thromboembolism in the intracranial circulation. Not treating the culprit lesion is associated with a potential risk of thrombus formation and stroke recurrence [7, 8]. Analyzed data from the German stroke registry, as well as the TITAN and ETIS registries demonstrated that recanalization of the ICA in TO leads to a significantly higher probability of successful reperfusion, better clinical outcome and significantly lower mortality [9, 10]. Acute CAS offers a one-stop treatment of the causative plaque and thus reduces the risk of reoccurrence [11]. However, such interventions are associated with the risk of in-stent thrombosis, ICH, and postprocedural ischemic events [4, 12]. The latter is attributed to the detachment of thrombus and plaque debris through the stent and occurs most likely when open-cell design [13] and not closed-cell design stents were used. Dual-layer/mesh stents (DLS) were designed in order to offer better plaque coverage and by this reducing periinterventional as well as secondary thromboembolic events. However, the great expectations placed on this new stent design were disappointed after several studies had reported high occlusion rates after the use of the dual-layer Casper-RX (Microvention, Tustin, CA, USA) in an emergent setting [14–16]. This discouraged interventionalists from implementing DLS in acute settings. Nevertheless, CGuard (InspireMD Inc., Tel Aviv, Israel) has a completely different design and has shown promising results in several multicentric trials in an elective setting [17, 18] whereas data on the use of the CGuard in acute CAS are very limited [19].

We aimed to compare the patency of the CGuard with the single-layer Carotid Wall-stent (CWS) in patients with acute symptomatic extracranial ICA occlusion or high-grade stenosis with or without TO, up to 72 h after the intervention.

## Methods

**Fig. 1:**
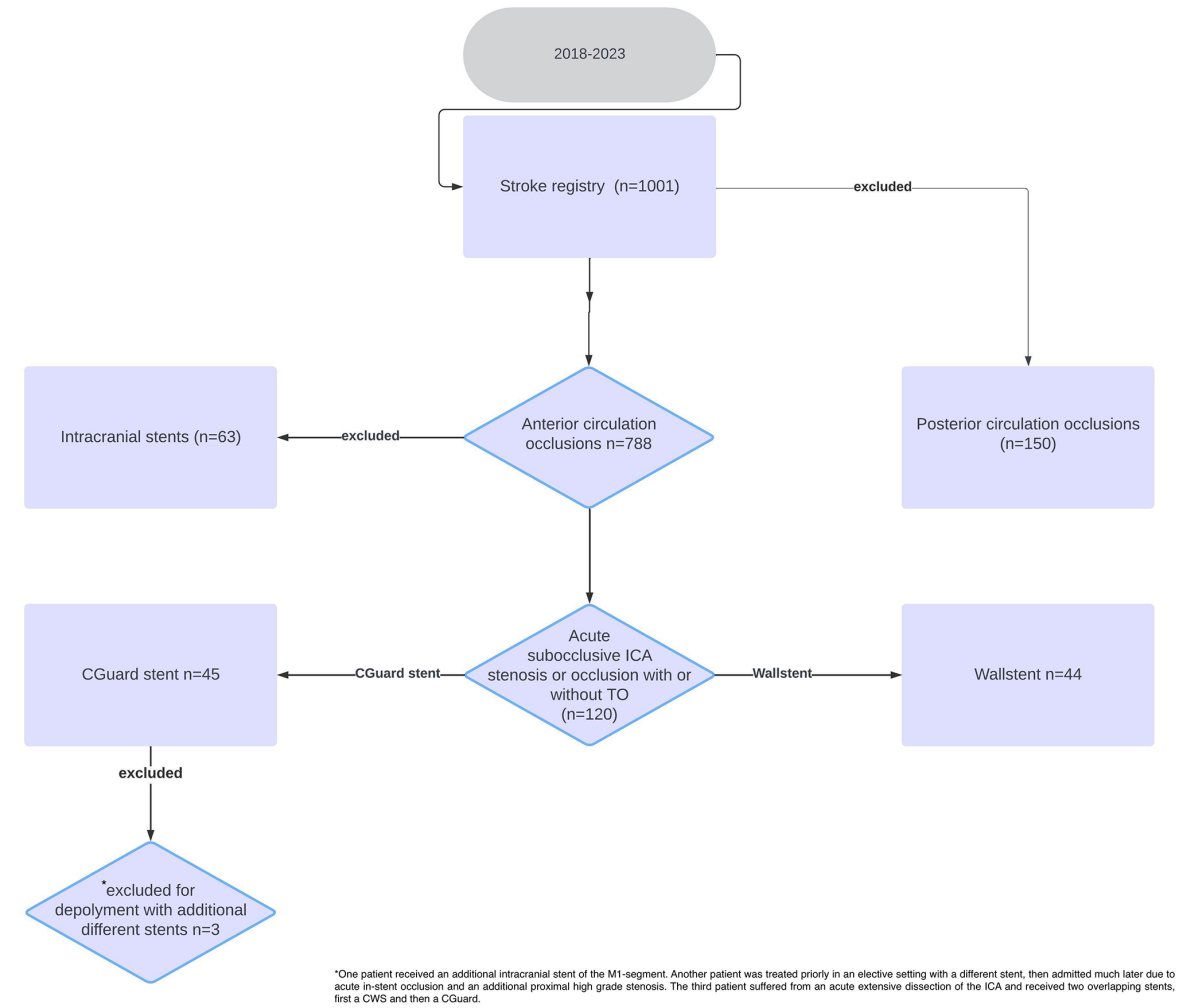
Represents a flowchart of the included patients from the comprehensive stroke center

Our prospective local thrombectomy registry was retrospectively analyzed for consecutive patients with AIS due to an acute symptomatic extracranial ICA occlusion or high-grade stenosis with or without TO, who received endovascular treatment between 11/2018 and 12/2022. In our comprehensive stroke center approximately 40% of MT patients are referred from other external hospitals and centers. Figure 1 represents a flowchart of the included patients from the comprehensive stroke center.

### Inclusion criteria

- Patients were referred for acute endovascular treatment and received emergent CAS using CGuard or CWS.
- Diagnosis was immediately established after computed tomography (CT) or magnetic resonance imaging (MRI) by the neuroradiologist.
- Patients showed a significant neurological deficit after undergoing the neurological examination per the National Institutes of Health Stroke Scale (NIHSS) score ≥ 4 before the intervention.
- Premorbid modified Rankin Scale (mRS) score ≤ 3.
- Achieved recanalization with a modified Thrombolysis in Cerebral Infarction (TICI) ≥ 2b.
- Patients who received follow-up imaging of the brain and the cervical arteries using CT, CT angiography, MR or Doppler ultrasonography to evaluate stent patency, detect intracranial hemorrhage and Infarction within 72 h after the intervention or earlier until discharge.

When indicated, intravenous r-tPA (recombinant tissue-type plasminogen activator) was administrated to eligible patients prior to endovascular therapy.

Research was conducted according to the principles of the declaration of Helsinki.

### Study device

The CGuard stent-system is a double-layered stent that consists of an inner open-cell nitinol stent and an outer closed-cell, single knitted polyethylene terephthalate (PET) mesh layer of 20 μm. It is a self-expandable stent delivered through a 6-F (2.0-mm) catheter [17]. The stent is available in sizes from 6 to 10 mm in diameter and lengths from 20 to 60 mm. The pore size of the mesh when the stent is fully deployed is 150 to 180 μm, with a free cell area of 16.25 mm^2^.

The CWS (Boston Scientific, Santa Clara, California) is a single-layer system consisting of cobalt-chromium alloy with closed-cell design and a free cell area of 1.09 mm^2^ [20]. Depending on stent size it is 5-F and 6-F sheath compatible.

**Fig. 2:**
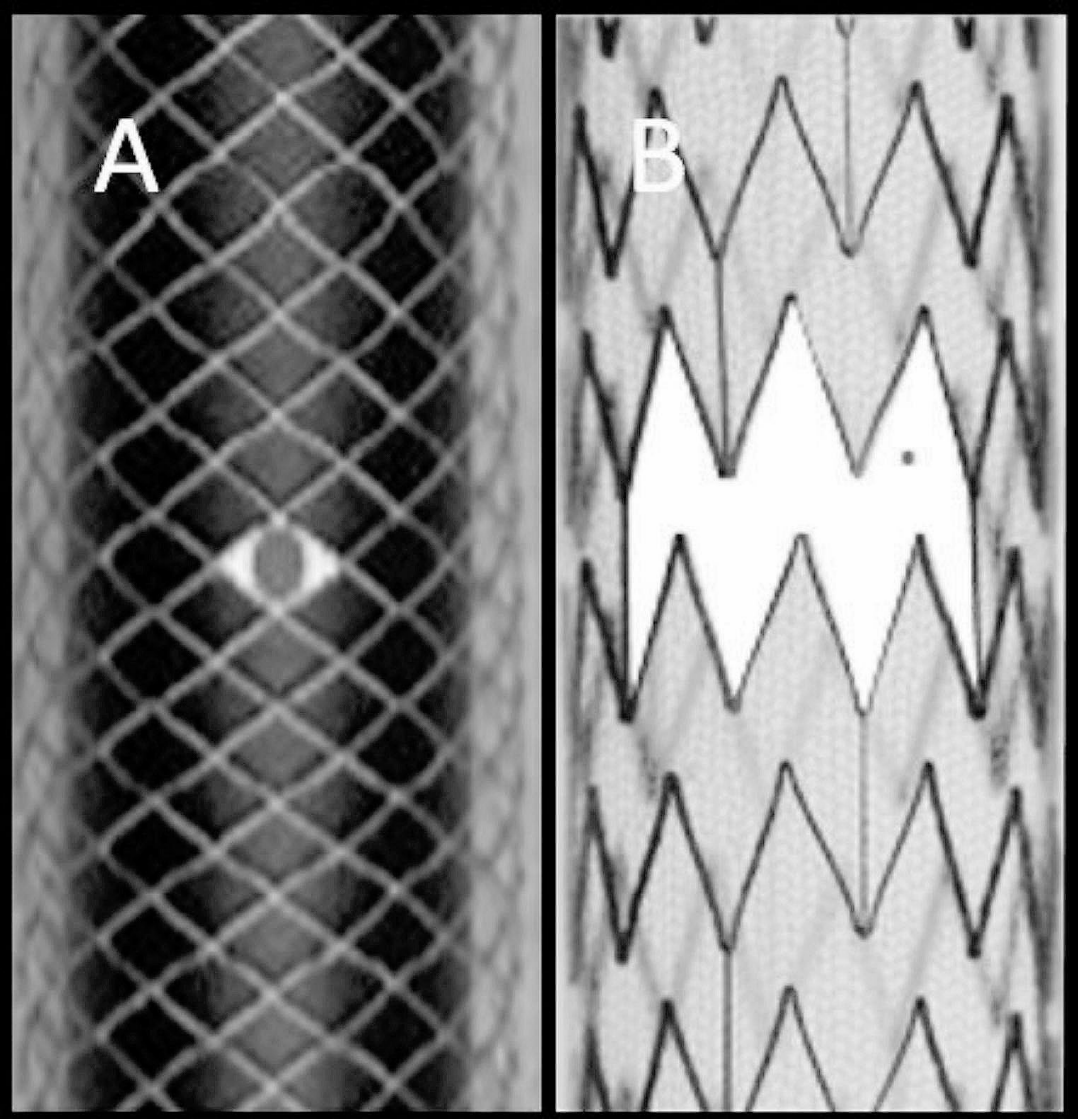
Illustration demonstrating the difference between the free cell size (white emphasized space) and pore size (grey emphasized space) between Wall-stent (**A**) and CGuard-stent (**B**). Free cell size offers flexibility and conformability during deployment, especially in torturous vessels. The pore size offers proper coverage of the underlying plaque

The differences between both stent designs are highlighted in Fig. 2.

### Endovascular procedures and peri-interventional antiplatelet/anticoagulation

All acute MT and CAS procedures were performed under general anesthesia via a transfemoral approach. Generally speaking, a short 8F-sheath was used and an 8F guiding catheter was placed in the proximal CCA with support of a 5F selective catheter. For passing the stenosis of the internal carotid artery or occlusion normally, a 0.014” microwire (Traxess, Microinvention, Aliso Viejo, CA, USA) was used. No distal protection devices were used in a setting of acute CAS. Further materials used were according to the judgment and experience of the neurointerventionalist. A retrograde approach (intracranial procedure before the CAS) was preferred and an antegrade approach was only chosen if the passage of the proximal occlusion was not possible otherwise. The indication for carotid artery stenting [with percutaneous transluminal angioplasty (PTA) as necessary] was given if a high-grade and hemodynamic stenosis remained after recanalizing the vessel, or if the risk of rapid re-occlusion was considered high due to the configuration of the lesion. Principally, all patients received initially intravenous heparin 3000 UI, and an additional 1000 IU for every additional hour during the intervention. Patients received i.v. acetylsalicylic acid ASA (500 mg) before stent-implantation. Per protocol after achieving intracranial recanalization and treating the culprit ICA lesion, an angiography of the intracranial circulation is performed to ensure that no new embolization to the intracranial circulation has occurred during the CAS. There were no constraints regarding the use of stent retrievers or aspiration maneuvers for MT of the intracranial occlusion if applicable, or different antiplatelet regimens and anticoagulants. In addition, there were no constraints regarding the succession of CAS with or without balloon angioplasty and MT. Periprocedural intravenous thrombolytic drugs were administered at the discretion of the treating neurologist and neuroradiologist.

### Peri- interventional antiplatelet therapy

Within 24 h after MT and CAS patients underwent follow-up with cranial CT to exclude significant intracranial hemorrhage. If no contraindications were detected dual-antiplatelet therapy (DAPT) was started. Platelet function test was performed with Multiplate testing to identify partial/non-responders.

### Outcome evaluation

NIHSS was evaluated at admission and discharge again by an experienced neurologist. To evaluate the infarct progression and whether intracranial hemorrhage is present, all patients received a CT or MRI within 24 h after the intervention. Intracranial hemorrhage was assessed according to the criteria of the European Cooperative Acute Stroke Study ECASS II [21]. The mRS was used as indicator of clinical outcome at discharge.

## Statistical analysis

Baseline patient characteristics were analyzed using descriptive statistics. We performed chi-squared tests for categorical variables and two-sided t-tests for continuous variables. Data are shown as mean with standard deviation (SD) or median with interquartile range (IQR). All calculations were performed using SPSS software (.

## Results

All patients with acute occlusions or high-grade stenosis of the extracranial ICA with or without middle cerebral artery (MCA) and/or intracranial ICA (carotid terminus) occlusions who were treated between November 2018 and December 2022 were identified from our stroke registry and were retrospectively analyzed. In this study, the first included CWS was used in November 2018 and the CGuard stent was first used in an emergent setting in November 2019 in our center. Three patients were excluded: one patient who underwent intra- and extracranial stenting, one patient who was treated with a CGuard stent after an acute in-stent-occlusion of another ICA stent, and one patient who was treated with a CWS for an acute dissection and with a CGuard after acute occlusion of the first stent. In addition, all patients with acute dissections who were treated with aspiration only or other stents/ flow diverters were not included in this study.

Overall, eighty-six patients were included: Forty-four patients were treated using the CWS and forty-two with the CGuard-stent. The underlying etiology of the high-grade stenosis of the ICA or occlusion was dissection in 5 patients (5.8%) and atherosclerosis in 81 patients (94.2%). The rates of the initial complete occlusions of the cervical ICA did not differ significantly between CWS- and CGuard-groups (59.1% versus 50%; *p* = 0.397). Balloon angioplasty prior to stenting was performed in both groups (40.9% vs. 35.7%, *p* = 0.620). Post-dilation was performed in both groups (22.7% vs. 19%, *p* = 0.537). All stents were patent on the final angiograms and no rest significant stenosis (> 50%) was detected after completion of the intervention. Mean age of patients in both groups was similar (CWS: 71.4, CGuard: 71.9) with a predominant number of male patients 70.5% and 69% respectively. Baseline median NIHSS score was 13 (IQR: 9–17) and 12 (IQR: 7–17) respectively with 45.5% and 47.6% of patients receiving IV tPA.

Baseline, demographic, interventional and imaging characteristics of the overall patient population are shown in Table 1.

**Table 1:** Clinical, radiographic, preprocedural, periprocedural and postprocedural characteristics of both patient groups

(Mean ± STD) [N], % (n/N) or median (IQR)	Wall-stent	CGuard stent	*P*-Value
*Age*	71.4 ± 12.6 [44]	71.9 ± 13.0 [42]	0.878
*Male*	70.5% (31/44)	69% (29/42)	0.887
*Female*	29.5% (13/44)	31% (13/42)	
***Comorbidities***			
*Hypertension*	75% (33/44)	73.8% (31/42)	0.899
*Diabetes mellitus*	45.5% (20/44)	33.3% (14/42)	0.25
*Dyslipidemia*	70.5% (31/44)	52.4% (22/42)	0.085
*Atrial fibrillation*	13.6% (6/44)	19% (8/42)	0.497
*Previous cardiovascular disease*	25% (11/44)	33.3% (14/42)	0.395
***Preprocedural***			
*Premorbid mRS*	0 (0–0)	0 (0–1)	0.615
*mRS at admission*	4 (4–5)	4(3–4)	0.13
*Baseline NIHSS*	13 (9–17)	12 (7–17)	0.622
*Baseline ASPECTS*	8 (7–9)	9 (7–9)	0.527
*IV t-PA use*	45.5% (20/44)	47.6% (20/42)	0.841
*Complete occlusion of the cervical ACI*	59.1% (26/44)	50% (21/42)	0.397
***Occlusion site***:			
*Intracranial ACI*	29.5% (13/44)	11.9% (5/42)	**0.044**
*M1-Segment*	56.8% (25/44)	50% (21/42)	0.526
*M2-Segment*	11.4% (5/44)	14.3% (6/42)	0.685
*Tandem*	86.4% (38/44)	73.8% (31/42)	0.144
***Procedural***			
*Balloon Angioplasty*			
*Predilation*	40.9% (18/44)	35.7% (15/42)	0.62
*Post-dilation*	22.7% (10/44)	19% (8/44)	0.537
***TICIs***			
*2b*	34% (15/44)	54.8% (23/42)	**0.033**
*3*	65.9% (29/44)	45.2% (19/42)	
***Complications***:			
*Intracranial hemorrhage*	20.5% (9/44)	16.7% (7/42)	0.652
*In-stent occlusion*	9.1%(4/44)	4.8% (2/42)	0.431
*In-stent stenosis*	2.3% (1/44)	2.4%(1/42)	0.973
***Clinical outcome***			
*mRS at discharge*	3(2–4)	2 (1–5)	0.285

NIHSS; National Institute of Health Stroke score, ASPECTS; Alberta Stroke Program Early CT Score, IV- t-PA; intravenous tissue plasminogen activator, ICA; internal carotid artery, MCA; middle cerebral artery, TICIs; thrombolysis in cerebral infarction scale. Values of *p* < 0.05 are considered statistically significant and are marked in bold

The rate of acute in-stent occlusions (occlusions within 72 h after stenting) was lower in the CGuard group when compared to the CWS group without statistical significance (*n* = 2; 4.8% vs. *n* = 4; 9.1%, *p* = 0.431). Three of the patients with acute in-stent occlusions (2 CWS and 1 CGuard) had dissection as an underlying pathology for the initial ICA occlusion. These occlusions occurred in less than 24 h postprocedural. The rest of the in-stent occlusions occurred between 48 and 72 h and each of those patients received DAPT postprocedural. Each group had one case of in-stent stenosis. CGuard patients performed slightly better with a discharge mRS of 2 (IQR:1–5) vs. 3 (IQR:2–4), *p* = 0.285, again without statistical significance. 52.4% of the CGuard group *n* = 22 had favorable outcomes at discharge (mRS ≥ 2) vs. CWS group *n* = 15, 34.1%, *p* = 0.087.

Patients received between 3000 and 8000 IU of heparin depending on the duration of the intervention. There was a statistically significant difference with regard to antiplatelet regimen administered after deployment of the stents (*p* < 0.001): CWS group received primarily Clopidogrel/ Acetylsalicylic acid (ASA) (75%), while the CGuard-group received predominantly Ticagrelor/ASA (83.3%).

The subgroup analysis of antiplatelet regimen among CWS patients did not reveal any significant differences regarding in-stent-occlusions or –stenosis. The same applies to the CGuard patients. Platelet inhibition testing didn’t recognize non- responders or impaired response to CPG among patients with in-stent occlusions. Intracranial hemorrhage was observed more often in the CWS-group without any statistical significance (*p* = 0.652; 20.5% vs. 16.7%). In addition, no significant association between the antiplatelet regimens and intracranial hemorrhage was found. Different antiplatelet regimens and frequency of ICH are presented in Table 2.

**Table 2:** Antiplatelet therapy administered to both patient groups and postprocedural hemorrhage

% (n/N)	Wall-stent	CGuard stent	*P*-Value
*Antiplatelet therapy*			**p < 0.001**
*Clopidogrel + ASA*	75% (33/44)	16.7% (7/42)	
*Ticagrelor + ASA*	13.6% (6/44)	83.3% (35/42)	
*Only ASA*	4.5% (2/44)	0% (0/42)	
*Only Clopidogrel*	6.8% (3/44)	0% (0/42)	
*Intracranial hemorrhage*			0.652
*HI1*	44.4% (4/9)	28.6% (2/7)	
*HI2*	22.2% (2/9)	28.6% (2/7)	
*PH1*	22.2.% (2/9)	28.6% (2/7)	
*PH2*	11.1% (1/9)	14.2% (1/7)	

HI; hemorrhagic infarct, PH; parenchymal hematoma. Values of *p* < 0.05 are considered statistically significant and are marked in bold

Only 15/42 (35.7%) of the CGuard group and 18/44 (40.9%) of the CWS-group received follow-up imaging at 6 months in our center. All CGuard stents were patent without stenosis. However, 6/18 (33.3) of the CWS group presented with in-stent stenosis and the difference between both groups was statistically significant (*p* = 0.039). Half of the CWS-patients with in-stent stenosis were retreated with balloon angioplasty. Stenosis grade and postprocedural antiplatelet therapy of the reported in-stent stenosis in the CWS-group are summarized in Table 3.

**Table 3:** Stenosis grade on follow-up imaging after 6 months in Wall-stent patients and administered postprocedural antiplatelet therapy

	Stenosis grade %	Postprocedural antiplatelet therapy	Retreatment
*Stenosis on follow-up at 6 months*	50% 70% 60% 60% 50% 50%	Ticagrelor + ASA Ticagrelor + ASA Clopidogrel + ASA ASA Ticagrelor + ASA Clopidogrel + ASA	No Yes Yes Yes No No

## Discussion

This investigation demonstrates that the deployment of CGuard stent was safe in the setting of emergent CAS. Compared to other reported dual-layer stents, the rate of acute in-stent-occlusions was not increased, but on the contrary lower when compared to the mono-layer CWS, however without statistical significance (4.8%vs. 9.1%). The rate of in-stent-occlusions after acute CAS with CGuard stent was lower in our series than in the single-center series including 33 patients ( 9% acute occlusion rate) reported by Klail et al. [22]. The rate of in-stent occlusions of CWS in our series was in range with the findings of previous studies [23]. However, this current acute in-stent-occlusion rate with the CGuard appears to be in direct opposition with the high occlusion rates with other DLSs (Casper-RX, Microvention) reported by Bartolini et al. (52.4%) [16]. Furthermore, Pfaff et al. reported in a multicentric study thrombus formation and complete occlusion of the Casper-RX during the procedure or within 72 h in 33/160 (20.8%) and in 12/160 patients (7.5%), respectively [15]. These alarming results lead to call from some authors for the discontinuation of DLSs [16].

For the purpose of analyzing the varying occlusion rates among different DLSs, it is important to understand the crucial differences in stent design. The Casper-RX stent is formed from a braided metal frame with nickel-titanium alloy mesh layer within the stent frame, whereas the protective, single-fiber knitted mesh in the CGuard-stent encompasses the frame of the stent from the outside. That means that CGuard stent has considerably less thrombogenic material and its outer mesh layer offers structural support. This can explain the previously reported higher rates of in-stent restenosis reported in other DLSs [24, 25]. The hybrid stent design in CGuard, meaning that the open cell component (free area 16.25 mm^2^ vs. 1.09 mm^2^ of CWS) offers flexibility and conformability especially in torturous vessels, something that has been limited for instance in closed-cell stents [26].

The rate of in-stent stenosis on the follow-up imaging at 6 months was higher in the CWS-group compared to the CGuard group (6 vs.0) and statistically significant (*p* = 0.039). One possible explanation is that the better coverage of the DLS prevents protrusion of the fresh thrombotic material more efficiently. However, due to the different antithrombotic regimes in this investigation and the low number of included follow-ups we are reluctant to overinterpret these results.

In the current investigation, post-dilation was considered when a restenosis > 50% occurred after deployment before the end of the intervention. There are several benefits and considerable risks related to post-dilation angioplasties. On one hand, it is thought to reduce the incidence of restenosis by re-establishing the normal diameter of the vessel through closely apposing the stent and intima [27]. However, several investigations have found that post-dilation increases the risk of silent ischemia and neurological events [28], as it presents a high risk for embolization when the balloon pushes the stent struts against the atheromatous plaque [29].

In this investigation, the underlying pathology for the acute occlusion or high-grade stenosis was predominantly atherosclerosis *n* = 81 (94.2%), in addition to 5 cases (5.8%) of acute dissection of the cervical ICA. Although, both represent very different pathologies. However, in clinical routine and in particular in an emergency setting it is often not easy to distinguish between both. Gory et al. did not find a significant difference between dissection and atherosclerotic tandem occlusions in the multi-centric evaluation of 295 patients (65 patients with a dissection) concerning clinical outcome, hemorrhage and mortality; however, there is no information on early re-occlusion in this cohort [30]. Similarly, it was found in the TITAN registry that the etiology of the cervical ICA lesion (atherosclerosis vs. dissection) did not impact the final reperfusion rates or clinical outcomes [31].

There was a significant difference between both groups with regards to number of accompanying intracranial ICA occlusions CWS 29.5% (*n* = 13) vs. 11.9% (*n* = 5) of CGuard. In total, CWS group included 4 cases of ICA-L and 9 of ICA-T occlusions, whereas all the intracranial ICA-occlusions in the CGuard group were ICA-T occlusions. ICA-T occlusions have been associated with a poor outcome as shown in previous observational thrombectomy studies [32, 33]. This may contribute to the fact that CWS patients had a worse clinical outcome post-procedurally.

There has been sufficient evidence that in-stent occlusions especially among patients with tandem occlusions after acute CAS stenting is significantly less frequent among patients receiving DAPT when compared to patients receiving mono antiplatelet therapy (MAPT) [34]. Neurointerventionalists face a conundrum. On one hand, dual antiplatelet therapy has been widely used to prevent in-stent thrombosis after aCAS [35]. On the other hand, pretreatment antiplatelet therapy before EVT is associated with an increased risk of ICH [36].

In this study, both stent groups received ASA during stent deployment followed by DAPT after the follow-up CT as a standard therapy for the prevention of in-stent occlusion [37]. The CWS group received predominantly ASA and clopidogrel, whereas the CGuard group received ASA and Ticagrelor. None of the DAPT variants were associated with an increased risk of in-stent occlusions excluding potential bias from this heterogeneity.

In our series, 3 out of 6 patients with an in-stent-thrombosis had an underlying dissection thus indicating that stent-implantation under monotherapy (no matter if it is the wall-stent or the CGuard) might be unsafe and should be potentially performed under a more aggressive antiplatelet regime, e.g. GPIIb/IIIa inhibitors or dual antiplatelets. However, this more aggressive medication may translate in an increased bleeding rate and must – at least before we have more reliable data - be carefully weighted in the individual case.

Furtherly, we did not find a higher rate of in-stent occlusion in patients who did not receive IV tPA in the current investigation, which is different from the findings reported by Yilmaz et al. [14].

Thus, we could not identify which DAPT is more thrombo-protective. This is relevant, because while Ticagrelor is potent, has a quicker onset than clopidogrel [38] and overcomes the issue of non-or poor-responders [39], its cost is significantly higher.

It is unfortunate in this investigation, that only 15/42 (35.7%) of the CGuard group and 18/44 (40.9%) of the CWS-group received follow-up imaging at 6 months. This is attributed to the high quota of external stroke patients referred to the current comprehensive stroke center for treatment, which are consecutively transferred back to their local hospitals and neurological rehabilitation centers after treatment.

### Limitations

There are several limitations: first, the collected data were from a single-center and analyzed retrospectively. The small number of observed occlusions in both patient groups introduce a severe data fragility. The retrospective nature of the study implies a number of limitations in the form of different patient characteristics, reported and unreported interventional technical aspects of the underlying procedures and heterogenous peri- and postinterventional medication regimes, as it was decided at the discretion of the treating neurologist and neuroradiologist, possibly influencing patient outcomes as well as the endpoints in-stent occlusion rate and intracranial hemorrhage rate. Second, missing further follow-up data prevent the assessment of stent patency mid- and long-term. Third, the modalities used to evaluate stent patency have varying sensitivity.

## Conclusions

We present the so far largest cohort of patients who were treated with the dual-layer CGuard-stent in an emergent setting, mainly in tandem occlusions. We were able to demonstrate that the in-stent occlusion rate was lower with the CGuard stent than reported with other dual-layer stents and comparable to the single-layered CWS. Further data are needed to evaluate the potential benefit of the design in more detail.

## Data Availability

Individual participant data that underlie the results reported in this article (after deidentification) will be available upon reasonable request. These proposals should be directed to the corresponding author.
